# Effects of a lotion containing probiotic ferment lysate as the main functional ingredient on enhancing skin barrier: a randomized, self-control study

**DOI:** 10.1038/s41598-023-43336-y

**Published:** 2023-10-06

**Authors:** Hongchang Cui, Congrui Feng, Tao Zhang, Verónica Martínez-Ríos, Patricia Martorell, Marta Tortajada, Sidao Cheng, Shumin Cheng, Zhi Duan

**Affiliations:** 1 Qingdao Vland Biotech Co., Ltd., 596-1 Jiushui East Road, Laoshan District, Qingdao, 266102 China; 2 ADM, Nutrition, Health and Wellness, Biopolis S.L., Parc Científic Universitat de València, C/ Catedrático Agustín Escardino Benlloch, 9, 46980 Paterna, Spain

**Keywords:** Microbiology, Health care

## Abstract

There is an emergent need to develop functional cosmetic ingredients for the topical management of skin barrier function. This study aimed to investigate the efficacy of a lotion containing fermented lysates VHProbi® Mix R for enhancing the skin barrier. In vitro studies demonstrated that fermented cultures of both *Lacticaseibacillus rhamnosus* VHProbi® E06 (E06) and *L. paracasei* VHProbi® E12 (E12) had antioxidant capacity, showing promising scavenging capability for 2,2-diphenyl-1-picryl-hydrazyl. The antioxidant capacity of these strains was also demonstrated in the model of *Caenorhabditis elegans*. In addition, the fermented lysates of both E06 and E12 enhanced the proliferation of HaCaT cells and ameliorated the toxicity induced by *Staphylococcus aureus* ATCC 25923, hydrogen peroxide, and ultraviolet B radiation in the HaCaT cell models, which simulated the irritants that facial sensitive skin is exposed to. Subsequently, the ingredient VHProbi® Mix R was formulated using four kinds of fermented lysates: E06, E12, *Lactiplantibacillus plantarum* VHProbi® E15, and *Lactobacillus helveticus* VHProbi® Y21. A clinical study was conducted to investigate whether a lotion containing VHProbi® Mix R would be beneficial for people to enhance skin barrier. The participants were asked to use the investigational product for 30 days. Several indicators, including transepidermal water loss (TEWL), skin moisturization, and redness were measured at day 0 and day 30 using VISIA®-CR and CK®-MPA systems. Meanwhile, the burden of sensitive skin (BoSS) and self-assessment questionnaires were performed at baseline and endpoint of this study. The study data showed that at day 30, there was a significant decrease in TEWL (*P* < 0.01), redness measured by CK®-MPA (*P* < 0.01), and redness profile measured by VISIA®-CR compared with the baseline measurements. Skin moisturization had significantly increased after treatment with the lotion for 30 days. BoSS and self-assessment questionnaires also substantiated that the participants felt a markedly positive change in their sensitive skin. Hence, we hypothesize that applying the topical functional VHProbi® Mix R could confer effective benefits for people with sensitive skin and this represents a promising intervention for enhancing skin barrier.

## Introduction

As the largest organ of the body, skin serves as the interface between the body and the external environment, exerting multiple pivotal protective functions against exposome aggressions, e.g., air pollution, climate stimuli, solar radiation, lack of sleep, malnutrition, etc.^1–4^. The status of the skin is an apparent signature reflecting the age and general health of the host, especially for exposed areas like the face. Some studies have demonstrated that a damaged skin barrier function is closely associated with the onset of sensitive skin^5–7^. When the skin barrier function is impaired, the permeation of harmful substances and the irritation of exposome can stimulate the Langerhans cells and keratinocytes to produce some mediators involved in the immune response, causing the symptom related to the sensitive skin^8,9^. Epidemiology studies show that a considerably increased incidence of sensitive skin in industrialized countries, with a prevalence of 60–70% in females and 50–60% in males^10–12^. Individuals with sensitive skin are prone to suffer contact dermatitis or irritations using skincare products^9,13,14^.

The impairment of skin barrier has a huge influence on the quality of life of individuals. Unfortunately, there is no simple regimen to enhance skin barrier function. In some cases, chemicals or steroids, including tacrolimus, trans-4-tert-butylcyclohexanol, phenoxyethanol, pimecrolimus, or corticosteroids, can be used to relieve the symptoms^15–17^. However, such treatment options are only for patients with severe symptoms, and are only available on prescription, and cannot be used long term. To date, although the prevalence of sensitive skin is increasing, there are limited publications focusing on safe, natural, and sustainable approaches to ameliorate the symptoms of impaired skin barrier.

An increasing number of evidence-based reports advance the hypothesis that certain probiotics are beneficial to the skin immune system, barrier function, and cutaneous microbiota, leading to a health status of skin homeostasis^18,18–20^. For instance, various of probiotic strains have been developed for applications in acne, atopic dermatitis, eczema, barrier repair, and so on^21–25^. Owing to the increasing popularity of the concept of probiotics, many consumers regard the application of probiotics as a promising and accessible approach to daily skin care. Indeed, some clinical trials have demonstrated that probiotic supplementation may contribute to the nourishment of impaired facial skin^26,27^. However, most studies concentrated on the nutraceutical ingredients, only few studies focused on the postbiotics to the preservation of impaired sensitive skin. The International Scientific Association of Probiotics and Prebiotics recently described postbiotics as “preparations of inanimate microorganisms and/or their components that confer a health benefit on the host”^28^. Using postbiotics on a host surface, including the skin and mucosa, has received support from scientific and cosmetic communities^29^. This highlights that using postbiotics as functional ingredients for topical skin care products may become more common.

In our previous study, we validated the efficacy of the fermented lysate from *Lactiplantibacillus plantarum* VHProbi® E15 (E15) in ameliorating the symptoms of acne^21^. Here, we developed a kind of fermented lysate designated VHProbi® Mix R that comprised four lactic acid bacteria including E15, *Lactobacillus helveticus* VHProbi® Y21 (Y21), *Lacticaseibacillus rhamnosus* VHProbi ® E06 (E06), and *Lacticaseibacillus paracasei* VHProbi® E12 (E12). In our preliminary investigation, E06 and E12 exhibited antimicrobial activity, oxidation resistance, and enhanced the skin barrier experiments conducted in vitro and in vivo. This study aimed to evaluate whether VHProbi® Mix R could confer beneficial effects in subjects with impaired skin barrier.

## Materials and methods

### In vitro studies

#### Bacterial strains and cells

The bacterial strains of E15and Y21 were isolated from kimchi. The bacterial strains of E06and E12 were isolated infant feces samples. All of strains were identified using the method described by Zhang and Duan^30^. The strains were harvested using de Man-Rogosa-Sharpe (MRS) agar medium (Luqiao, Beijing, China) under aerobic atmosphere at 37 °C for overnight. *Staphylococcus aureus* ATCC 25923 was purchased from the American Type Culture Collection (Manassas, VA, USA) and maintained on a Tryptic Soy Broth medium (TSB; Hope Bio-Technology, Qingdao, China) at 37 °C for 24 h under stationary conditions.

To obtain the fermented lysate, bacterial strains were inoculated in fermentation medium (2% molasses, 0.1% peptone, 5% collagen, 0.3% (NH_4_)_2_HPO_4_, and 1000 mL distilled water) and incubated at 37 °C for 48 h. The fermentation was then homogenized using a homogenizer (APV-1000, SPXFLOW, USA) under 1000 bar and heated at 70 °C for 10 min before being used in subsequent experiments.

A human keratinocyte cell line, HaCaT cells, purchased from BeNa Culture Collection (Beijing, China), was grown in Dulbecco’s modified essential medium (DMEM; Sigma-Aldrich, USA) supplemented with 10% heat-inactivated fetal bovine serum (FBS; Sigma-Aldrich, USA) and incubated at 37 °C in a 5% CO_2_ atmosphere. Cells were seeded into a sterile 24-well plate (Corning, NY, USA) and were used when reaching 80% confluence.

#### Scavenging capability of E06 and E12 for 2,2-diphenyl-1-picryl-hydrazyl (DPPH)

The antioxidation assay was conducted using the method described by Sanchez-Moreno et al. with slight modification^31^. Briefly, 1 mL of ethanolic DPPH solution (0.4 mM) was vigorously mixed with 1 mL fermented culture (intact cells > 10^9^ CFU/mL) or vehicle (control) and incubated under dark conditions at 37 °C for 30 min. The optical density (OD) of the solution was then measured at 517 nm using a microplate reader (Multiskan SkyHigh, ThermoFisher Scientific, USA). Scavenging activity was calculated as: scavenging capacity % = [1 − (A_sample_ − A_blank_) /A_control_] × 100%, where A_sample_, A_blank_, and A_control_ is the absorbance of the sample, the blank (ethanol and cells), and the control (vehicles), respectively.

#### Oxidative stress resistance in Caenorhabditis elegans

*Caenorhabditis elegans* wild-type strain N2 were age-synchronized in nematode growth medium (NGM) plates (control) and NGM plates containing E06 and E12 bacterial strains at final dose of 10^8^ CFU/plate. Nematode’s viability was assessed after an acute oxidative stress (2 mM H_2_O_2_) as described by Martorell et al.^32^. Vitamin C (10 µg/mL) was used as positive control. Experiments were carried out in duplicate.

#### Proliferation assay for Lysate on HaCaT cells

HaCaT cells were plated at 2 × 10^5^ cells/well in a 24-well plate and incubated for 24 h prior to the proliferation assay. Next, the HaCaT cells were co-incubated with the lysate of E12 and E06 at a multiplicity of infection (MOI) 10 and 100 at 37 °C in a humidified incubator under 5% CO_2_ for 24 h. The cells were treated with MTT (3-(4, 5-dimethylthiazol-2-yl)-2, 5-diphenyltetrazolium bromide, Abcam) with a final concentration of 0.3 mg/mL and incubated at 37 °C for 3 h. Untreated cells were served as a control. The MTT solutions were discarded and DMSO was added to solubilize the MTT-formazan crystals. Finally, the OD of cells was measured at 490 nm^33,34^.

#### Cell viability assay for protective effect of lysate

To evaluate the protection effect of E06 and E12 on HaCaT cell viability, *S. aureus* ATCC 25923, H_2_O_2_ (Merck, USA), and ultraviolet-B (UVB) radiation were used to induce the deterioration of cell viability. Briefly, HaCaT cells were prepared described as above. For the *S. aureus*-induced damage model, cells were treated with 1μL of intact cells of *S. aureus* ATCC 25923 (10^8^ CFU/mL), then lysates of E06 and E12 were added individually at a MOI of 10 and 100. The mixture was incubated at 37 °C under 5% CO_2_ conditions for 16 h. After incubation, the supernatant was measured by using a Lactate Dehydrogenase Cytotoxicity Assay Kit (Cat: C0017, Beyotime, Shanghai, China)^35^.

For the H_2_O_2_-induced damage model, cells were treated with the lysates of E06 and E12 individually at a MOI of 10 and were incubated at 37 °C for 3 h. Subsequently, peroxide solutions were added to the mixture with a final concentration of 0.5 mM and were incubated for 1 h. Next, the medium was discarded and washed twice in PBS (Sigma, USA), then incubated at 37 °C under 5% CO_2_ conditions for 16 h. After incubation, the cells were treated with MTT and measured using the same method as described in the proliferation assay.

For the UVB radiation-induced damage model, cells were treated with the lysates of E06 and E12 individually at a MOI of 100 and were incubated at 37 °C for 3 h. Next, the cells were treated with UVB radiation system (LONGPRO, Shanghai, China) using the dose of 60 mJ/cm^2^ at the spectrum of 313 nm. After irradiation, the MTT test was performed using the method described in the proliferation assay^36,37^.

### Clinical studies

#### Lotion formula

The ingredients of the lotion were: 3% VHProbi® Mix R, 4% propylene glycol, 4% caprylic/capric triglyceride, 4% mineral oil, 3.5% cetearyl alcohol, 1.5% PEG-100 glyceryl stearate, 1.5% stearic acid, 0.6% glyceryl stearate, 0.5% 1,2-hexanediol, 0.3% phenoxyethanol, 0.2% xanthan gum, 0.15% chlorphenesin, % L-arginine, and 100 mL aqua. The VHProbi® Mix R was formulated using the lysate of E06, E12, E15, and Y21. These contents, except for VHProbi® Mix R, were used as preservatives, chelating agents, and thickeners in the lotion.

#### Subjects

A total of 52 eligible adult subjects, including males and females, were enrolled after verification of the criteria (Table 1). This trial was conducted by Qingdao Yisu Biotech Ltd located in the No.66 Huazhong Road, High-tech Zone, Qingdao, 266100, China. All subjects provided their written informed consent to participate the study and the publication of identifying information or images. Ethical approval was not necessary based on the Safety and Technical Standards of Cosmetics (2015 version) and Specification for the Cosmetic Efficacy Claim Evaluation issued by the National Medicine Product Administration in China^38,39^. All methods in the study are in accordance with Declaration of Helsinki. Subjects with a skin phototype classification of I – IV according to the Fitzpatrick scale^40^, and who were confirmed to have sensitive skin based on a positive response (prickling, itching, burning, stinging with an intensity score ≥ 2) to the lactic acid stinging test^41^, and a BoSS (Burden of Sensitive Skin) questionnaire total score ≥ 8, were included in the study^42^. The detailed information of all subjects is presented in Table S1. In terms of controlling other confounders, we completed the enrollment of subjects between June and August to exempt the effects of season on the results. During recruitments, two of staffs were responsible for managing the inclusion and exclusion of the participants to harmonize the subjects. And the ombudsman was responsible for supervise the process to ensure the compliance. Data entry, cleaning and analysis was conducted by two staffs to ensure the authenticity, accuracy and completeness of data.

**Table 1:** Inclusion/exclusion criteria for the clinical study.

Inclusion criteria	Exclusion criteria
Female or male > 18 years old	Known sensitivity to any compound of the lotion
Provided informed consent	Pregnancy or breastfeeding
Neither currently nor in the past 6 months participated in the same study	Facial cosmetic procedures within 3 months or facial disease
Comply with the requirements from investigators and back to study center on timely	Cosmetic product use within 21 days that could interfere with the study
Last treatment was at least half year ago	Heavy psychological and psychiatric disorders that could lead the subjects not to meet the study requirement
Non responsive to phototherapy, topical, or systemic	History use of immunosuppressants, local anesthetics, nonsteroidal, anti-inflammatory drugs, antihistamines, or corticosteroids within 14 days or during this study
Not receiving any cosmetic/ cosmeceutical treatments that might interfere with current study	

#### Study procedures and assessments

The subjects applied an equal amount to 0.6–0.8 g per time of the investigational lotion twice daily to their face for 30 days. After 15 min of resting in a room, the subjects were asked to complete the BoSS questionnaire on the repairing effect of the sensitive skin barrier. The dermatology assessments, including transepidermal water loss (TEWL), moisturization, and redness were conducted using the S0083 TEWL Probe, S0033 Moisture Measurement Probe, and S0043 Melanin Measurement Probe of CK-MPA® system (CK-MPA10, Courage + Khazaka electronic GmbH, German), respectively. Probes were lightly and quickly pressed on the surface of skin. The evaluation of redness profile indicator that was calculated by evaluating the dense and red areas integrated was performed by using the VISIA®-CR system (Canfield Scientific Inc., NJ, USA). The indicators described above plus BoSS (Table S3) and self-assessment questionnaire were investigated at baseline (Day 0) and endpoint (Day 30) of the study^42^.

### Statistical analysis

Data were initially analyzed by EpiData ver3.1 and exported to SPSS ver26.0 (SPSS Inc., USA) for further analysis. Data distribution was assessed by the 1-sample Kolmogorov–Smirnov test. Parametric values are expressed as means ± standard deviations. The Wilcoxon test or paired samples t- test for paired samples was performed to evaluate changes between the data obtained at baseline and outcome data. *P* < 0.05 and *P* < 0.01 were considered to be significant and highly statistically significant difference, respectively. Graphics were produced by using GraphPad Prism ver8.0.

### Ethical statement

Ethics review and approval was not required for this study on the human participants in accordance with the local legislation and institutional requirements. The participants provided their written informed consent to participate this study. Written informed consent was obtained from individuals for the publication of any potentially identifiable images or data included in this study.

## Results

### In vitro studies

#### DPPH radical scavenging activity of E06 and E12

The DPPH free radical scavenging activity of E06 and E12 is shown in Table 2. The fermented cultures of these two strains had relatively higher antioxidation capabilities, with a scavenging rate of 38.22% for E06 and 34.44% for E12.

**Table 2:** DPPH radical scavenging activity.

Strain	Scavenging rate (%)
E06	38.22 ± 2.21
E12	34.44 ± 4.74

Means ± standard deviation.

#### Antioxidant effect of E06 and E12 in C. elegans

**Figure 1:**
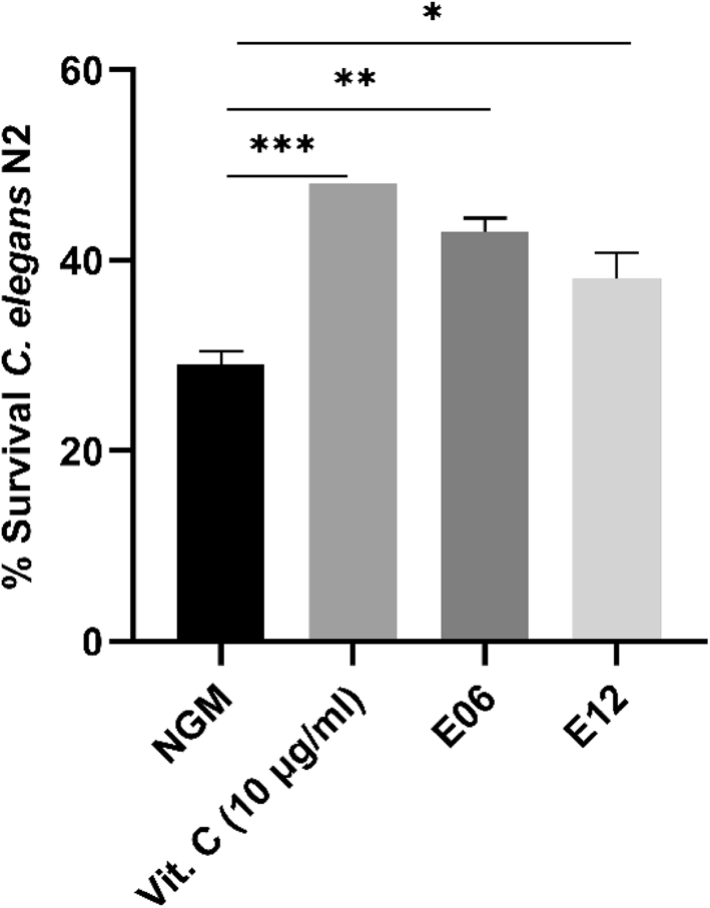
Survival of *C. elegans* (N2) fed by E06 and E12 after acute oxidative stress. NGM: Nematode growth medium used as control feeding condition. ***P < 0.001; ** P < 0.01; *P < 0.05 compared with control (NGM).

Survival of *C. elegans* after an acute oxidative stress is shown in Fig. 1. Nematodes fed with probiotics E06 and E12 were significantly more resistant to H_2_O_2_ oxidative stress than control-fed nematodes (29% NGM vs. 43% E06, 38% E12).

#### Enhancing proliferation effect of lysate on keratinocytes

**Figure 2:**
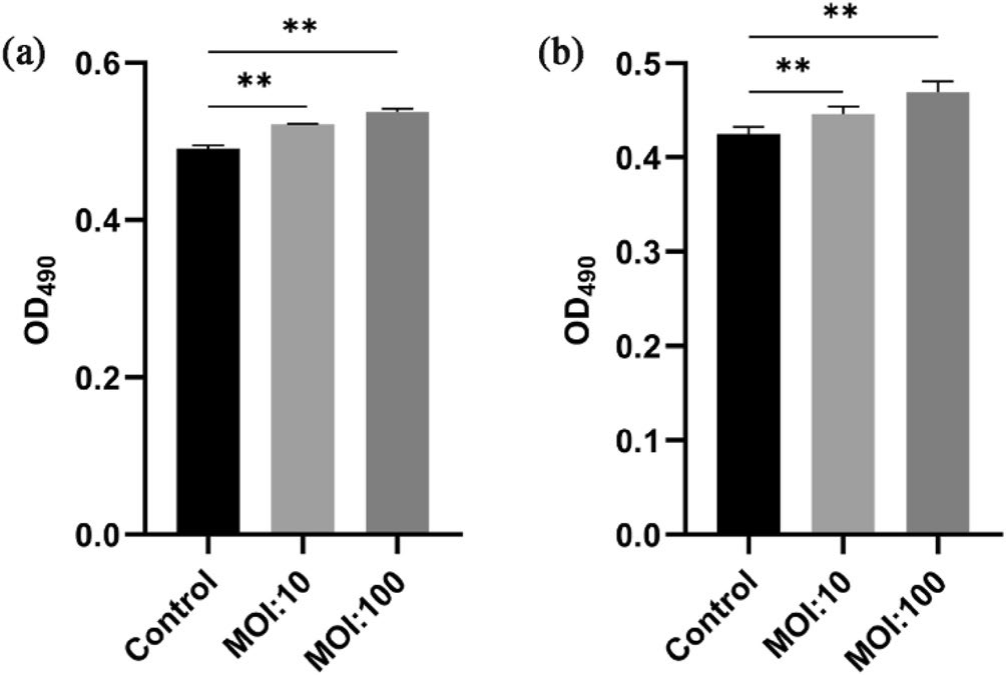
Absorbance variations of HaCaT Cells treated with or without lysate. (**a**) Proliferation-enhancing effect of lysate E06 on HaCaT Cells. (**b**) Proliferation-enhancing effect of lysate E12 on HaCaT Cells. Control: HaCaT Cells treated without lysate; MOI: multiplicity of infection. **P < 0.01 compared with control.

The ability of E06 and E12 to enhance the proliferation of HaCaT cells is shown in Fig. 2a,b, respectively. The absorbance at 490 nm indirectly represents the viability of HaCaT cells. The mean values of absorbance for control, MOI:10, and MOI:100 of lysate E06 were 0.49, 0.52, and 0.54, respectively (Fig. 2a). This indicates regard less of whether the MOI value was 10 or 100, the HaCaT cells exhibited significantly higher viability compared with the control (*P* < 0.01), in which HaCaT cells were not co-incubated with the lysate of E06. The mean values of absorbance for control, MOI:10, and MOI:100 of lysate E12 were 0.42, 0.45, and 0.47, respectively (Fig. 2b). This also indicated that, the HaCaT cells had significantly enhanced viability compared with the control after treatment with the lysate of E12, at an MOI of 10 or 100. Thus, it can be concluded that the fermented lysates of E06 and E12 provided proliferation- enhancing effects on HaCaT cells.

#### Protective effect of lysate on HaCaT cells

**Figure 3:**
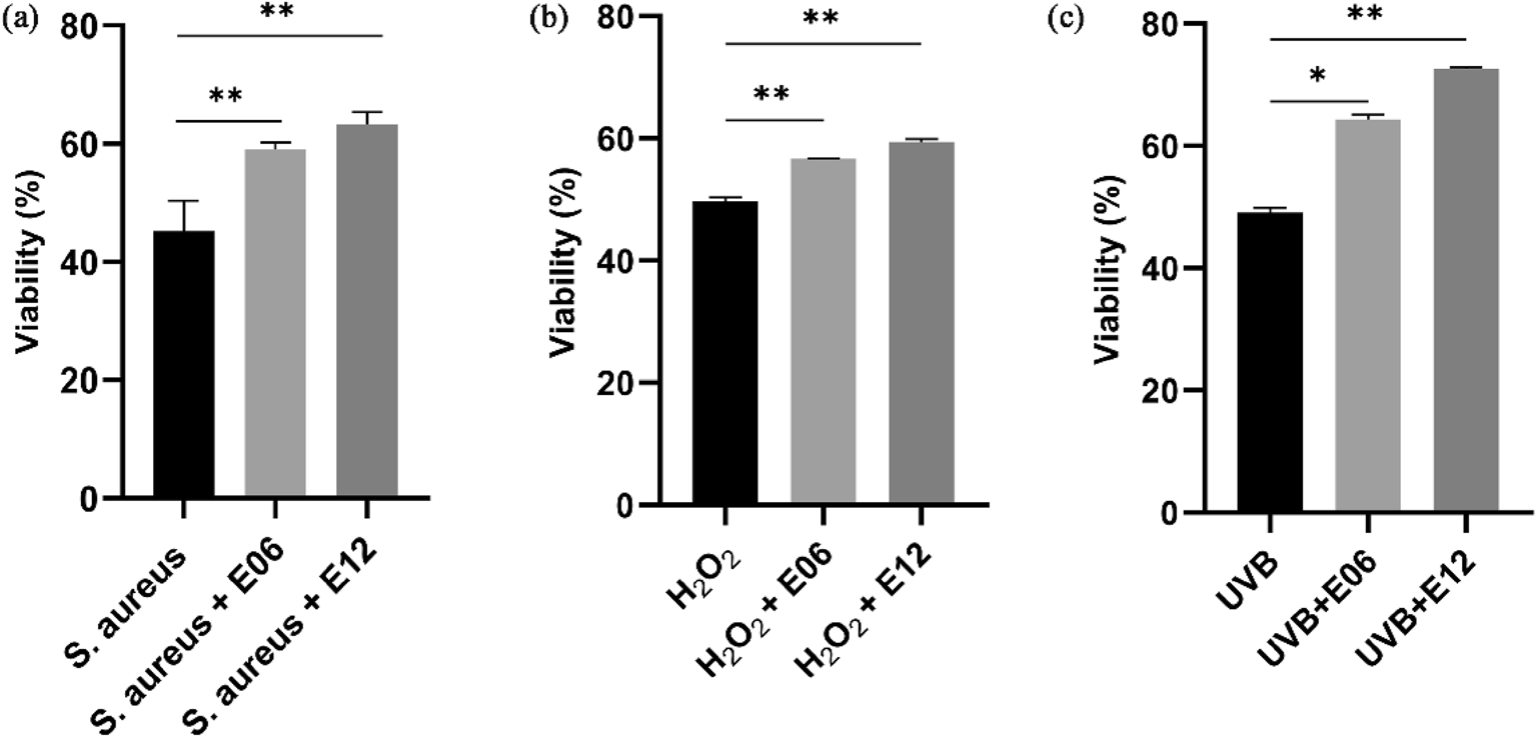
Protection of the lysate on HaCaT cells against different stimulants. (**a**) Viability rates of cells treated with *S. aureus* alone or with lysates. (**b**) Viability rates of cells treated with H_2_O_2_ alone or with lysates. (**c**) Viability rates of cells treated with UVB alone or with lysates. *S. aureus*: *S. aureus* ATCC 25923; UVB: ultraviolet B; *P < 0.05. **P < 0.01.

To determine the protective effect of the lysates on the viabilities of HaCaT cells, three different stimulants, including *S. aureus* ATCC 25923, hydrogen peroxide solution, and UVB radiation, were used to induce damage to HaCaT cells. The viability rate of cells was 45.23% after co-incubation with *S. aureus*, whereas the cells exhibited significantly higher viability rates when treated with *S. aureus* plus the lysates of E06 and E12, reaching 59.06% (*P* < 0.01) and 63.32% (*P* < 0.01), respectively (Fig. 3a). Hence, the viability rates increased by 13.83% and 18.09% with the lysates E06 and E12 compared with cells treated with *S. aureus* alone, respectively. As shown in Fig. 3b, the viability rate of cells was 49.79% after treatment with H_2_O_2_ alone, but this increased to 56.66% and 59.43% when cells were treated with H_2_O_2_ combined with the lysate of E06 and E12, respectively, and these differences were statistically significant (*P* < 0.01). The cells displayed significantly lower viability after treatment with the UVB radiation in comparison with those treated with radiation combined with the lysates (Fig. 3c). Specifically, the viability rate of cells in the UVB + E06 group was 64.34%, which was significantly higher than that of the UVB group (49.12%, *P* < 0.05). Cells from the UVB + E12 group also exhibited significantly higher viability compared with those of the UVB group, reaching 72.59% (*P* < 0.01). Collectively, these data demonstrated that the lysates of E06 and E12 decreased the toxicity of different stimulants to HaCaT cells, providing protective effects on viabilities of keratinocytes against common facial skin damage.

### Clinical studies

#### Detection of TEWL

**Figure 4:**
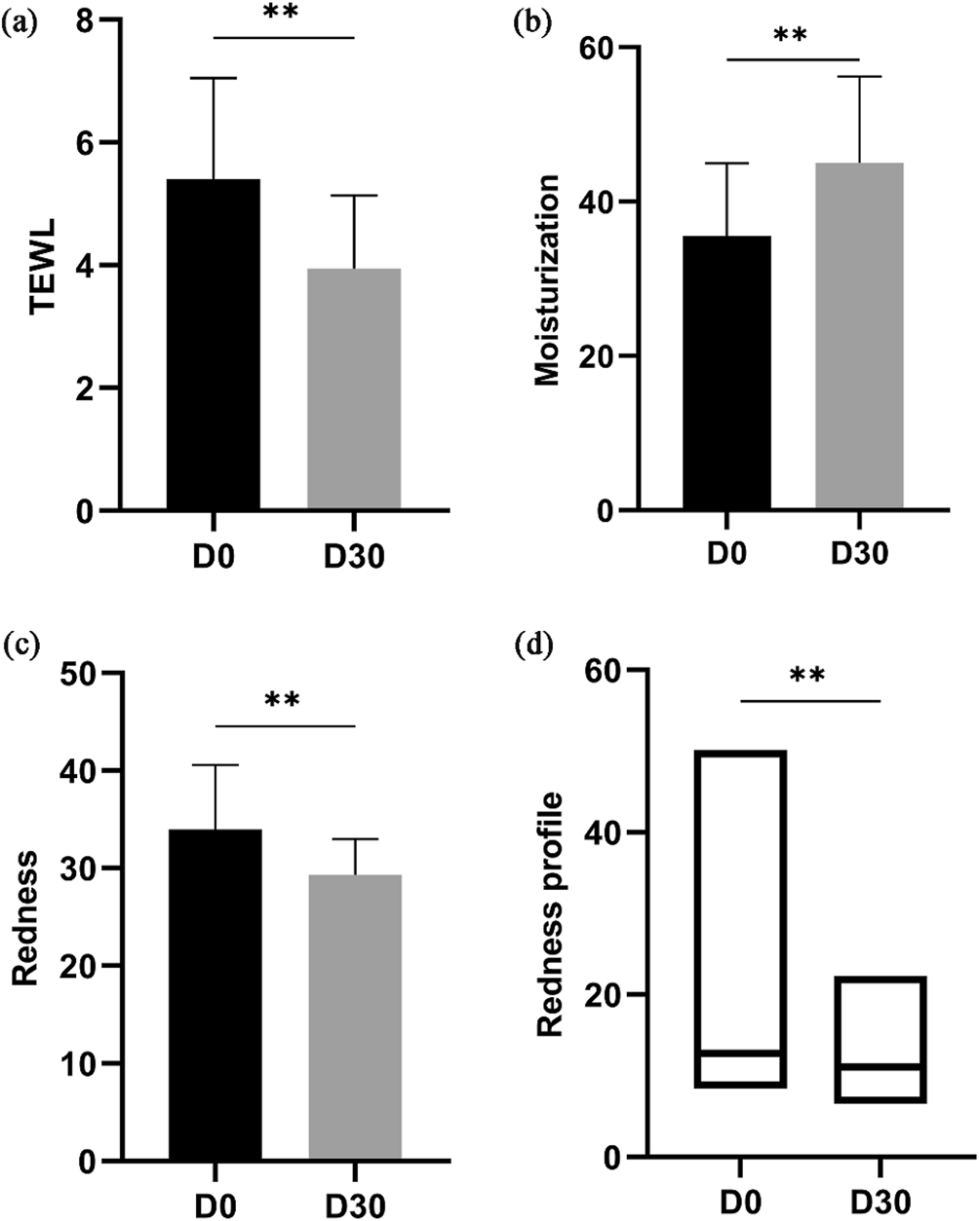
Improvement of cutaneous statuses related to sensitive skin. (**a**) TEWL, transepidermal water loss. (**b**) Moisturization. (**c**) Redness. (**d**) Redness profile. D0: day 0; D30: day 30; **P < 0.01.

Detailed scores on the TEWL indicator for individuals from baseline to the final visit are presented in Table S2. As shown in Fig. 4a, the mean value of TEWL declined from 5.38 at day 0 to 3.94 at day 30, a decreased of 26.8%. This decrease was statistically significant (*P* < 0.01), suggesting that the lotion containing VHProbi® Mix R may provide a protective effect in reducing skin water loss.

#### Skin moisturization

Detailed scores on moisturization for individuals from baseline to the final visit are presented in Table S2. As shown in Fig. 4b, the mean value of skin moisturization increased by 26.6% from 35.58 at day 0 to 45.02 at day 30. This difference in the moisturization indicator at the study endpoint compared with that at baseline was statistically significant (*P* < 0.01). Thus, it may suggest that the lotion containing VHProbi® Mix R can improve the water retention capacity of skin.

#### Skin redness

**Figure 5:**
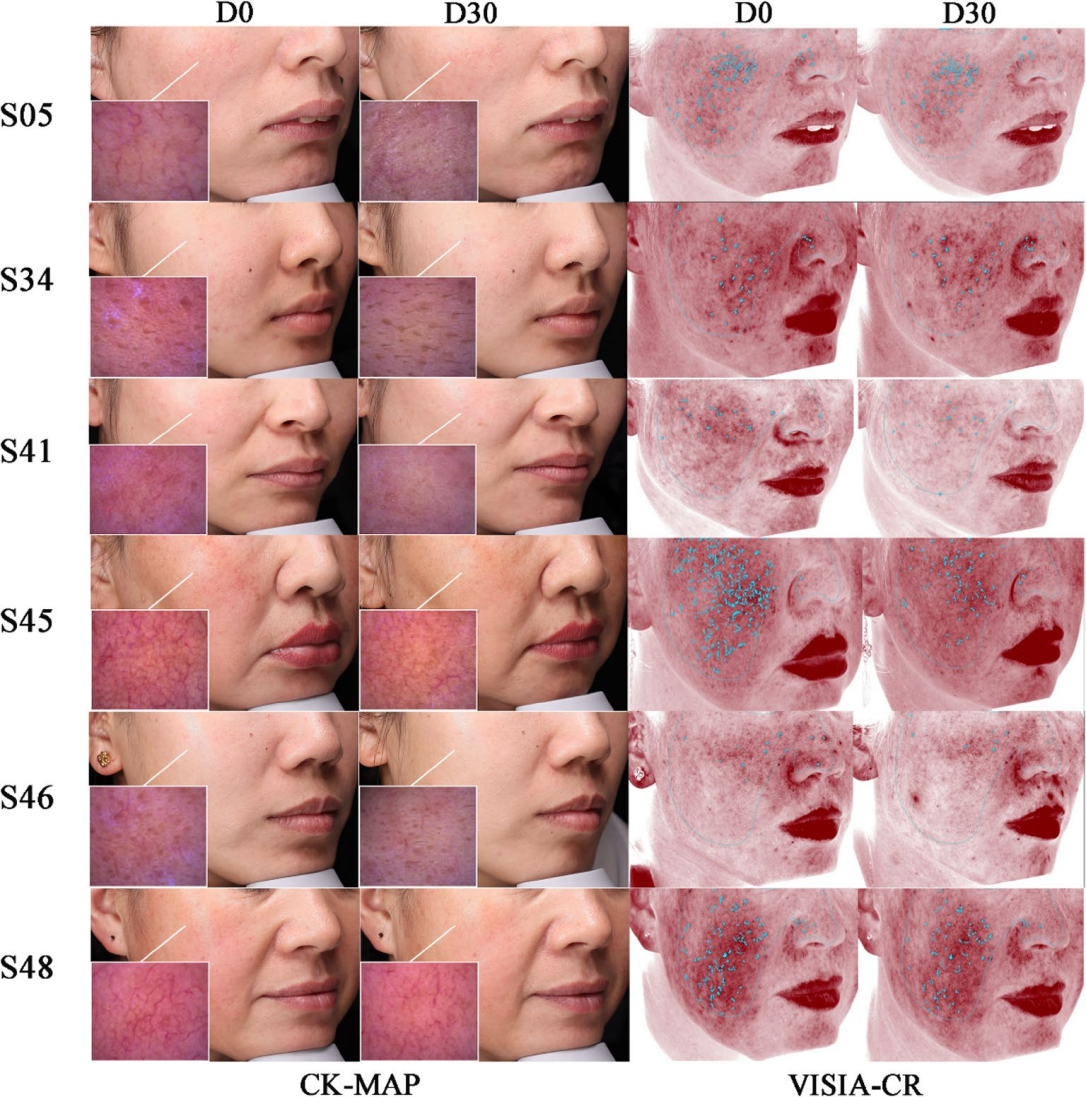
Microscopic and macroscopic images of skin redness area captured under cross-polarized light using CK and VISIA instruments. D0: day 0; D30: day 30.

Detailed scores on skin redness for individuals from baseline to the final visit are presented in Table S2. As shown in Fig. 4c, the mean value of skin redness decreased from 33.98 at day 0 to 29.30 at day 30. This decrease of 13.8% in skin redness from baseline to endpoint was statistically significant (*P* < 0.01). The representative improvement in the skin redness of the subjects can be observed through the microscopy images taken under cross-polarized light (Fig. 5, CK). A decreased trend in terms of vascularity, the dull red area, and the dense and thick spider veins was intuitively observed after treatment with the investigational product for 30 days.

#### Skin redness profile

Detailed scores on redness profile for individuals from baseline to the final visit are presented in Table S2. As shown in Fig. 4d, the median values (interquartile range) of skin redness profile value decreased by 17.1% from 12.79 (10.92–16.14) to 11.08 (9.95–14.12), after 30 days of treatment, which was a statistically significant difference (*P* < 0.01). The efficacy of the VHProbi® Mix R in ameliorating severity of redness from a macroscopic perspective was also confirmed by using the VISIA®-CR system (Fig. 5, VISIA). The indigo stars represent the concentrated darker red area, which may be related to some kinds of inflammation and spider veins, and showed improved characteristics at the end of study.

#### BoSS and self-assessment questionnaire

In terms of BoSS questionnaire (see Table 3), the mean total score decreased from 23.29 to 17.63 after 30 days of treatment with investigational product containing VHProbi® Mix R. At the endpoint, the total score decreased by 24.3%, reaching a statistically significant difference in comparison with the baseline (*P* < 0.01). The indicator of self-care means individuals take risk factors into consideration at the decision-making circumstances such as buying cloths and cosmetics. The indicator of daily life represents the lifestyle such as avoiding irritants. The appearance simply indicates the facial characters such as prone to be red. These three indicators significantly decreased at the end of study (*P* < 0.01), with reductions of 11.9%, 32.5%, and 30.9%, respectively. The BoSS questionnaire elucidated that the subjects had a significantly reduced burden of sensitive skin after using the investigational product for 30 days.

**Table 3:** BoSS questionnaire of skin parameters.

Parameters	Time	Mean ± SD	Mean change from baseline ± SD	*P*	% Change from baseline
Self-care	D0	8.75 ± 5.71	1.04 ± 0.79	< 0.01	11.9
	D30	7.71 ± 5.40			
Daily life	D0	7.33 ± 2.46	2.38 ± 0.99	< 0.01	32.5
	D30	4.94 ± 2.13			
Appearance	D0	7.21 ± 2.42	2.23 ± 1.29	< 0.01	30.9
	D30	4.98 ± 2.10			
Total score	D0	23.29 ± 7.90	5.65 ± 1.53	< 0.01	24.3
	D30	17.63 ± 7.82			

D0: day 0; D30: day 30; SD: standard deviation; *P* < 0.01 indicates a significant difference.

The self-assessment questionnaire also illustrated that the VHProbi® Mix R may play an important role in improving skin quality (see Table S4). More than 80% of the subjects reported that using the test products can mitigate many skin problems such as xerosis, tightness, the local inflammation, scaling, itching, tingling or pain, the stability of skin with change in emotion, stress, or temperature. In addition, 73% of the subjects reported a good or excellent improvement in skin redness and 75% of the subjects reported a good or excellent reduction in skin impurities such as swelling, oozing, scabs, and rashes.

## Discussion

The impairment of skin barrier function is a common cutaneous disorder raising challenges for dermatologists, patients, and the cosmetic industry. Increasing advancements in elucidating the mechanism of action for strengthening skin barrier provide promising interventions for the management of these individuals^43^. In recent years, an enormous variety of so-called hyposensitive skin care products has emerged, which assert to soothe numerous signs of sensitive skin. However, none of existing standard guidelines for skin care formulations refer to the complexity of managing individuals with damaged skin barrier^44^. To date, skin barrier repair products have become a huge commercial market, reaching to numerous billions per year. Consumers are increasingly paying attention to their consumption footprint and consciously seeking personal care products containing natural and clean ingredients^45^. Another popular trend is that with the ingredients available for internal and external applications to address skin health and inflammatory responses, pro- and postbiotics show great potential in the personal care space^46^.

In this study, in vitro experiments substantiated that the fermented cultures of E06 and E12 each displayed promising DPPH scavenging capacities. The investigational strains can produce many antioxidative agents including superoxide dismutase, glutathione peroxidase, multivitamins, and etcetera after fermentation. DPPH is a kind of free radical. It is probably that the scavenging agents existing in fermented cultures reacted with the DPPH. Furthermore, using *C. elegans* as simple in vivo model confirmed the ability of both strains to increase resistance to oxidative stress. This animal model has become an excellent model to assess the antioxidant properties of probiotics, postbiotics and ingredients both in topical or oral administration. In addition, the lysates of E06 and E12 can both improve the decreased viability of HaCaT cells caused by H_2_O_2_. Excessive free radicals damage cell components and devitalize enzymes, causing tissue injury through lipid peroxidation^47^. The balance between pro-oxidant and antioxidant substances is closely correlated with health status in the body. The excessive production of reactive oxygen species such as H_2_O_2_ may lead to the oxidation of polyunsaturated fatty acids and tissue damage^48,49^. We postulate that VHProbi® Mix R containing the lysate of both E06 and E12 can deliver antioxidant activity, serving as an ingredient for sensitive skin management.

Furthermore, the lysates of both E06 and E12 both enhanced the proliferation of HaCaT cells, and the promotion of proliferation was closely correlated with the concentration of the lysate. The lysates of E06 and E12 also conferred a protective effect on HaCaT cells, ameliorating the loss of cell viability caused by *S. aureus* and UVB radiation. HaCaTs cells are long-lived, spontaneously immortalized, human keratinocyte line that are able to differentiate in vitro, and are a useful model to investigate repair inventions/therapies on skin disease^50^. Increased proliferating capability of the HaCaT cells provides fundamental roles in the repair of sensitive skin. *S. aureus* is a common component of human skin microbiota and capable of causing various diseases of skin and skin structure disease^51^. Many epidemiological studies have demonstrated that UV exposure induces significant changes in the immune system and is regarded as a major risk factor in the development of sensitive skin^52–54^. In this study, damage to HaCaT cells induced by *S. aureus* ATCC 25923 or UVB irradiation was significantly prevented by addition of the E06 and E12 lysates. Coupled with the proliferation enhancement effect of the lysates, we propose that the lysate could function as a protectant against environmental stress-induced irritants that can lead to impaired skin barrier.

Subsequently, we performed a clinical study to investigate whether the lotion containing VHProbi® Mix R could strengthen the skin barrier function or not. The water retention capacity of skin, including the TEWL and moisturization, was markedly improved following treatment with the investigational product. The indictor of TEWL represents the water loss of skin through epidermis. The decrease of TEWL indicates the recovery of skin barrier integrity. And the skin moisturization indicates the water content of skin stratum corneum. A better recovery of TEWL would lead to an increase of skin moisturization. An impaired skin barrier function is mostly associated with the xerosis, or “dry” skin, giving rise to an increase in TEWL^55^. Our study substantiated that the lotion containing VHProbi® Mix R could repair skin barrier function in the individuals with sensitive skin. Meanwhile, the severity of redness area significantly declined, as confirmed by two measurements using CK® and VISIA® systems, respectively. Moreover, BoSS and self-assessment questionnaires provided verification from another angle that the subjects felt an improvement in the discomfort of sensitive skin.

In recent years, topical postbiotic applications have attracted considerable attention from scientific and industrial communities. Gueniche et al. conducted an in vitro study and a human trial using *Bifidobacterum longum* extract and demonstrated that the bacterial lysate could be developed as a new approach for the treatment and/or prevention of symptoms associated with the sensitive skin such as decrease in vasodilation, edema, TNF-α release, and mast cell deregulation^27^. Khmaladze et al. performed a comparative study on the effect of probiotics and lysates on human skin proved that live bacteria and the lysate of *Limosilactobacillus. reuteri* DSM 17938 reduced the level of proinflammatory cytokines such as IL-6 and IL-8 in a UVB radiation-induced inflammatory model, demonstrating that *L. reuteri* DSM 17938 could be beneficial for general skin health to improve skin barrier^25^. A similar phenomenon was observed when we used a lotion containing VHProbi® Mix R in the daily management of sensitive skin for 30 days. We hypothesize that the lysates combination, VHProbi® Mix R, could be developed as a new cosmetic ingredient for improving skin barrier function.

The field of postbiotics is continuously evolving. Increasing clinical and experimental research provides evidence that postbiotics can not only exert healthy effects on gastrointestinal function, but can also be used for dermal application owing to their distinctive properties. Given these aspects, the development of postbiotics for topical applications represents a promising treatment and/or intervention for the management of skin barrier function. However, there are some limitations in this study, including the absence of a control group and placebo effects in the clinical trial, no elucidation of the skin microbiota variations in the subjects, and only a relatively short duration of the intervention was examined. We acknowledged that the rigorous evidence may not be obtained from this study due to lacking the placebo group. Some positive changes for the subjects using the investigational products from baseline to the endpoint of the study would lay foundation for us conducting a randomized, placebo control trail.

## Conclusion

The results from this study implied that the lotion containing VHProbi® Mix R fermented lysate is well tolerated in strengthening skin barrier and may be able to mitigate the severity of sensitive skin after 30 days of intervention. Detailed immunomodulatory studies are needed to determine whether the components of VHProbi® Mix R could influence the pro-inflammatory and anti-inflammatory immune response, thereby affecting the development of sensitive skin. Further randomized, placebo controlled clinical studies are also required to validate the findings here and to better understand the mechanisms of postbiotics in the topical management of common dermatological conditions.

### Supplementary Information

Supplementary Tables.

## Data Availability

The original contributions presented in this study are included in the article/supplementary materials, further inquiries can be directed to the corresponding author.
