# Supplementary material for: Effects of a lotion containing probiotic ferment lysate as the main functional ingredient on enhancing skin barrier: a randomized, self-control study
Source: Sci Rep. 2023 Oct 6;13:16879. doi: 10.1038/s41598-023-43336-y (PMC10558477; doi:10.1038/s41598-023-43336-y)
Supplement: Supplementary file 1 — Supplementary Tables. [file 41598_2023_43336_MOESM1_ESM.docx]

Supplementary Material

Table S1. Characteristics of the study subjects

| Number | n=52 |
| --- | --- |
| Age | 35.38±10.349 |
| Men, n (%) | 1 (1.9%) |
| Women, n (%) | 51 (98.1%) |

Note: Age value represents by means ± standard deviation.

Table S2. Detailed scores of indicators related to sensitive skin

|  | TEWL | | Moisturization | | Redness | | Redness profile | |
| --- | --- | --- | --- | --- | --- | --- | --- | --- |
|  | Day 0 | Day 30 | Day 0 | Day 30 | Day 0 | Day 30 | Day 0 | Day 30 |
| S01 | 3 | 2 | 28 | 34.3 | 25 | 33.7 | 16.237 | 12.374 |
| S02 | 8 | 4 | 38 | 52.3 | 38.3 | 34.3 | 14.988 | 12.054 |
| S03 | 8 | 1 | 48 | 56.3 | 31.3 | 25.3 | 10.548 | 10.024 |
| S04 | 5 | 4 | 36.7 | 40 | 31 | 19.7 | 12.789 | 11.42 |
| S05 | 9 | 8 | 25 | 37.7 | 29 | 24.3 | 22.026 | 19.063 |
| S06 | 1 | 2 | 35 | 41.7 | 25 | 23.7 | 11.744 | 9.822 |
| S07 | 4 | 4 | 35.3 | 52.3 | 30.7 | 29.3 | 10.328 | 10.007 |
| S08 | 6 | 4 | 31.3 | 48 | 39.3 | 27.7 | 12.272 | 11.271 |
| S09 | 5 | 3 | 58.7 | 47.7 | 44 | 21 | 17.115 | 13.827 |
| S10 | 3 | 1 | 29.3 | 62 | 29 | 23.7 | 9.924 | 9.717 |
| S11 | 5 | 5 | 35.3 | 28 | 41.3 | 40.3 | 10.106 | 8.544 |
| S12 | 5 | 3 | 50.5 | 44 | 24.3 | 34 | 11.223 | 10.605 |
| S13 | 5 | 3 | 40 | 45.7 | 25.7 | 20 | 12.505 | 12.489 |
| S14 | 1 | 1 | 39.3 | 51.7 | 25 | 26 | 12.859 | 9.778 |
| S15 | 4 | 3 | 47.7 | 43.7 | 38.7 | 32.7 | 10.389 | 8.174 |
| S16 | 7 | 5 | 38.3 | 36.7 | 24 | 24 | 11.466 | 9.846 |
| S17 | 4 | 4 | 34 | 52.3 | 30 | 26.3 | 8.415 | 8.238 |
| S18 | 7 | 4 | 43.3 | 44.7 | 36.7 | 31.7 | 15.098 | 13.042 |
| S19 | 2 | 2 | 23.3 | 38.7 | 36 | 34.7 | 15.832 | 14.929 |
| S20 | 8 | 6 | 33.3 | 35 | 45 | 34 | 27.248 | 22.28 |
| S21 | 20 | 19 | 53.7 | 64.3 | 36 | 18 | 12.145 | 10.768 |
| S22 | 5 | 3 | 41 | 35.7 | 33.3 | 31.7 | 9.138 | 8.806 |
| S23 | 5 | 5 | 28.7 | 49 | 31.7 | 28.3 | 15.701 | 15.373 |
| S24 | 8 | 7 | 37.7 | 45.7 | 46.7 | 34 | 12.492 | 10.418 |
| S25 | 3 | 2 | 23.3 | 39.7 | 38.3 | 23.7 | 10.432 | 10.073 |
| S26 | 14 | 4 | 36 | 44 | 34.3 | 23.7 | 18.049 | 12.427 |
| S27 | 4 | 6 | 50.3 | 24 | 30 | 19.7 | 14.928 | 10.885 |
| S28 | 5 | 4 | 20.7 | 30.3 | 40 | 35.3 | 19.979 | 15.891 |
| S29 | 5 | 4 | 38.7 | 66.7 | 21 | 21.7 | 8.651 | 6.504 |
| S30 | 5 | 4 | 29.3 | 36 | 31.7 | 23.3 | 14.275 | 12.786 |
| S31 | 7 | 7 | 33.7 | 65.7 | 46 | 43.7 | 24.857 | 22.197 |
| S32 | 3 | 2 | 22.7 | 33.3 | 27 | 19.7 | 15.068 | 14.212 |
| S33 | 1 | 1 | 22.3 | 25.7 | 34 | 28.7 | 10.38 | 9.928 |
| S34 | 9 | 7 | 18 | 46 | 42.3 | 35.7 | 15.241 | 13.232 |
| S35 | 9 | 6 | 49 | 24.7 | 51 | 35 | 26.743 | 20.254 |
| S36 | 3 | 3 | 38.7 | 58.3 | 29.3 | 34 | 11.472 | 9.792 |
| S37 | 6 | 4 | 27.7 | 36.3 | 28.3 | 36.7 | 16.445 | 14.927 |
| S38 | 3 | 4 | 48.7 | 29 | 28.7 | 26.3 | 9.17 | 7.725 |
| S39 | 10 | 9 | 32.7 | 60.7 | 38.3 | 32.3 | 13.401 | 10.894 |
| S40 | 5 | 4 | 28.7 | 38.3 | 21 | 19 | 11.187 | 10.296 |
| S41 | 4 | 1 | 49.3 | 52 | 34 | 31 | 12.787 | 11.443 |
| S42 | 3 | 2 | 35 | 56.7 | 28.7 | 24.3 | 10.83 | 10.081 |
| S43 | 4 | 2 | 23 | 45.3 | 33.7 | 32.3 | 13.835 | 11.985 |
| S44 | 4 | 1 | 38.7 | 53.3 | 46.7 | 52 | 23.323 | 21.527 |
| S45 | 3 | 1 | 50.7 | 57.3 | 42 | 42 | 50.135 | 17.676 |
| S46 | 7 | 4 | 30.7 | 50.7 | 30.7 | 21 | 11.679 | 10.542 |
| S47 | 7 | 5 | 30.3 | 42.3 | 31 | 25.3 | 11.412 | 10.199 |
| S48 | 3 | 2 | 31.7 | 63 | 45 | 39.3 | 26.914 | 19.31 |
| S49 | 3 | 2 | 34 | 34.7 | 32.3 | 24.3 | 11.194 | 10.541 |
| S50 | 4 | 4 | 38.7 | 34.7 | 32.7 | 27 | 13.296 | 12.437 |
| S51 | 1 | 1 | 25.3 | 59 | 40 | 41 | 9.797 | 7.112 |
| S52 | 7 | 6 | 30.7 | 46 | 31.7 | 27.3 | 20.041 | 17.305 |

TEWL: transepidermal water loss.

Table S3. **The BoSS questionnaire for sensitive skin**

| Please answer questions below | Constantly | Often | Sometimes | Rarely | Never |
| --- | --- | --- | --- | --- | --- |
| I have to consider my sensitive skin when I buy my clothes and underwear. | 🞎 | 🞎 | 🞎 | 🞎 | 🞎 |
| I have to consider my sensitive skin when 1 buy cosmetics. | 🞎 | 🞎 | 🞎 | 🞎 | 🞎 |
| Having sensitive skin slops me from eating certain foods. | 🞎 | 🞎 | 🞎 | 🞎 | 🞎 |
| I have given up hobbies, outings and holidays because of my sensitive skin. | 🞎 | 🞎 | 🞎 | 🞎 | 🞎 |
| I find it hard to tolerate air conditioning because of my sensitive skin. | 🞎 | 🞎 | 🞎 | 🞎 | 🞎 |
| I find it hard to cope with urban pollution because of my sensitive skin. | 🞎 | 🞎 | 🞎 | 🞎 | 🞎 |
| Blushing for no reason or after I become emotional embarrasses me when 1 am with other people. | 🞎 | 🞎 | 🞎 | 🞎 | 🞎 |
| My face often looks very red in photographs so 1 avoid appearing in them. | 🞎 | 🞎 | 🞎 | 🞎 | 🞎 |
| It is impossible for me to wear jeweler (bracelets, neck chains or chain bracelets) that is not made of gold. | 🞎 | 🞎 | 🞎 | 🞎 | 🞎 |
| My face turns red when I exercise, walk quickly or climb stairs. | 🞎 | 🞎 | 🞎 | 🞎 | 🞎 |
| Wearing woolen clothes close to my skin is unbearable. | 🞎 | 🞎 | 🞎 | 🞎 | 🞎 |
| I have to consider my sensitive skin when 1 choose my clothes in the morning. | 🞎 | 🞎 | 🞎 | 🞎 | 🞎 |
| I have to choose my own washing powder because some washing powders can cause a skin reaction. | 🞎 | 🞎 | 🞎 | 🞎 | 🞎 |
| When I sleep away from home, 1 take my own soap and toiletries with me because I cannot use other people's. | 🞎 | 🞎 | 🞎 | 🞎 | 🞎 |

Table S4. **The results of** **self-assessment questionnaire for sensitive skin**

| **Please answer efficacy-related questions below** | **Not at all** | **Slightly** | **Some** | **Good** | **Excellent** |
| --- | --- | --- | --- | --- | --- |
| Do you feel your skin more hydrated? | 0(0.0%) | 1(1.9%) | 6(11.5%) | 13(25.0%) | 32(61.5%) |
| Do you feel your skin less tight? | 0(0.0%) | 0(0.0%) | 8(15.4%) | 18(34.6%) | 26(50.0%) |
| Does your skin have less scaling? | 0(0.0%) | 0(0.0%) | 6(11.5%) | 22(42.3%) | 24(46.2%) |
| Do you feel your skin less itchy? | 0(0.0%) | 0(0.0%) | 7(13.5%) | 19(36.5%) | 26(50.0%) |
| Do you feel your skin less stinging or painful? | 0(0.0%) | 0(0.0%) | 8(15.4%) | 25(48.1%) | 19(36.5%) |
| Does your skin have less redness? | 1(1.9%) | 0(0.0%) | 13(25.0%) | 19(36.5%) | 19(36.5%) |
| Does your skin become more stable with the change of emotion, stress or temperature? | 0(0.0%) | 2(3.8%) | 5(9.6%) | 18(34.6%) | 27(51.9%) |
| Do you find your skin with less local inflammation? | 0(0.0%) | 1(1.9%) | 7(13.5%) | 22(42.3%) | 22(42.3%) |
| Do you feel other improvement in discomforts or show impurities? (Impurities including Swelling、Oozing、Scabs、Rashes、etc.)? | 0(0.0%) | 3(5.8%) | 10(19.2%) | 23(44.2%) | 16(30.8%) |

Note: Values are number (percentage).
